# Association of veteran suicide risk with state-level firearm ownership rates and firearm laws in the USA

**DOI:** 10.1136/ip-2023-045211

**Published:** 2024-09-30

**Authors:** Andrew R. Morral, Terry L. Schell, Adam Scherling

**Affiliations:** 1 RAND Corp Washington Office, Arlington, Virginia, USA; 2 RAND Corporation, Santa Monica, California, USA

**Keywords:** Suicide/Self?Harm, Firearm, Community

## Abstract

**Background:**

Veterans have higher suicide rates than matched non-veterans, with firearm suicides being especially prevalent among veterans. We examined whether state firearm laws and state firearm ownership rates are important risk factors for suicide among veterans.

**Methods:**

US veteran’s and demographically matched non-veteran’s suicide rates, 2002–2019, are modelled at the state level as a function of veteran status, lethal means, state firearm law restrictiveness, household firearm ownership rates and other covariates.

**Results:**

Marginal effects on expected suicide rates per 100 000 population were contrasted by setting household firearm ownership to its 75th versus 25th percentile values of 52.3% and 35.3%. Ownership was positively associated with suicide rates for both veterans (4.35; 95% credible interval (CrI): 1.90, 7.14) and matched non-veterans (3.31; 95% CrI: 1.11, 5.77). This association was due to ownership’s strong positive association with firearms suicide, despite a weak negative association with non-firearm suicide. An IQR difference in firearm laws corresponding to three additional restrictive laws was negatively associated with suicide rates for both veterans (−2.49; 95% CrI: −4.64 to –0.21) and matched non-veterans (−3.19; 95% CrI: −5.22 to –1.16). Again, these differences were primarily due to associations with firearm suicide rates. Few differences between veterans and matched non-veterans were found in the associations of state firearm characteristics with suicide rates.

**Discussion:**

Veterans’ and matched non-veterans’ suicide risk, and specifically their firearm suicide risk, was strongly associated with state firearm characteristics.

**Conclusions:**

These results suggest that changes to state firearm policies might be an effective primary prevention strategy for reducing suicide rates among veterans and non-veterans.

**WHAT IS ALREADY KNOWN ON THIS TOPIC**

- Veterans have higher suicide rates than matched non-veterans, and these higher rates are associated with greater firearm suicides. We examined whether state firearm laws and state rates of household gun ownership were risk factors associated with suicide risk among veterans and matched non-veterans.

**WHAT THIS STUDY ADDS**

- We found that restrictive state firearm laws and low state household firearm ownership are associated with lower total and, specifically, firearm suicide rates among veterans and matched non-veterans.

**HOW THIS STUDY MIGHT AFFECT RESEARCH, PRACTICE OR POLICY**

- Community firearm characteristics determined in part by state and local policies are risk factors for firearm suicide among veterans and non-veterans. Interventions to modify these factors should be further investigated as candidate suicide prevention measures.

## Background

 Suicide rates among veterans are higher than among non-veterans. They are especially high among some subgroups of veterans, such as those under the age of 45 for whom suicide was the second leading cause of death in 2021.^1^

Military experiences or exposures that might explain veterans’ elevated suicide rates have been extensively studied. Such experiences may contribute to veterans having firearm ownership rates—a risk factor for suicide^2^—approximately twice those of non-veterans.^3^ Similarly, risk factors for suicide have been examined in service members’ military trauma,^4^ military sexual trauma,^5^ traumatic brain injuries associated with military service,^6^ military deployments,^7^ stressors associated with transitioning from military to civilian life,^8^ interactions between psychopathology and military service^9^ and other features of military service.

Risk factors unrelated to military service have received less consideration in the study of veteran suicide risk. These include factors known to be associated with suicide risk in the general population. State of residence is one such risk factor, with suicide risk varying by a factor of three across states.^10^ This variation has been associated with state demographics,^11^ welfare policies,^12^ minimum wage laws,^13^ firearm and alcohol availability and firearm laws,^14^ among other state differences.

Veterans are more likely than non-veterans to die in suicides using a firearm. In 2020, 72% of veteran suicides involved a firearm, compared with 52% of non-veteran suicides^1^ and firearm suicide rates in the general population vary by a factor of 10 across states. For example, over 2018–2021, Massachusetts had 2 firearm suicides per 100 000 population, whereas Wyoming had 21 per 100 000.^10^ Differences of these magnitudes suggest that state of residence may be a more important risk factor for suicide than many of the individual-level characteristics that have been well studied, such as male gender which has been found to have an OR of 2.2, severe depression (OR=2.2), alcohol and drug misuse (OR=2.2), comorbid disorders (OR=1.6) or family histories of psychiatric disorder (OR=1.4).^15^

In this study, we investigate state differences in suicide risk among veterans. Specifically, we examine the association of state household firearm ownership rates and state firearm laws with veterans’ suicide rates, and whether these associations are different than found for matched non-veterans. In the general population, household firearm ownership rates and state suicide rates are highly correlated (eg, r=0.7 for all suicides and r=0.8 for firearm suicides^16^). Similarly, many studies examining the association of firearm regulations with state suicide rates have suggested that restrictive state firearm laws, like safe storage laws, waiting periods and minimum age of purchase or possession laws, are associated with fewer suicides and firearm suicides.^14^

Whether state firearm characteristics are associated with veterans’ suicide risk in a similar manner as found in the general population is not known. If they are then these potentially modifiable risk factors might point toward individual-level, community-level and policy interventions as effective or more effective than interventions more specifically targeted to veteran populations. However, veterans’ military experience, firearm ownership rates and increased likelihood of settling in areas other than where they grew up^17^ may all mitigate the influence of local community firearm ownership rates and state firearm laws, suggesting their suicide rates may be less associated with their states’ firearm characteristics.

## Methods

### Suicides and population data

Veteran suicide statistics were provided by the Veterans Affairs Administration (VA) Office of Mental Health and Suicide Prevention (OMHSP). This office collects identifiers on all current and former service members drawing from multiple data sources including paper service records prior to 1974. These identifiers are then matched to the National Death Index. Decedents are classed as veterans if they served on federal active duty and were not currently serving at the time of their death.^18^ These records omit some veteran suicides that did not occur in a US state, and some that occurred among veterans who served before electronic personnel records were implemented by the U.S. Department of Defense (DoD) in 1974.^19^ Nevertheless, they are the most comprehensive and well-vetted source of veteran suicide data available.^20^ OMHSP provided RAND with a public use data extract from this data set consisting of veteran suicide counts by state and firearm/non-firearm method, and by state and age of death, for six 3-year periods beginning in 2002 and ending in 2019. This file included veteran population counts by state and period from the VetPop model maintained by the VA’s National Center for Veterans Analysis and Statistics. Microdata and more detailed tables on veterans’ suicide are not publicly releasable due to privacy concerns.

### Non-veteran suicides and population data

Firearm and non-firearm suicides in each state and 3-year period were obtained for male and female decedents in three age groups (25–45, 46–64 and 65 and older) from the Centers for Disease Control (CDC),^10^ which also provided population estimates for each group. Suicide counts and population estimates were weighted to match the estimated age and gender distribution of veterans in each state and period (see supplemental methods in the online supplemental materials). Non-veteran suicides and population counts were estimated as the difference between the weighted total suicide counts and veteran suicides as provided in the VA data for each state and time period.

### Patient and public involvement

No patients were involved in this study.

### Predictors

**Figure 1:**
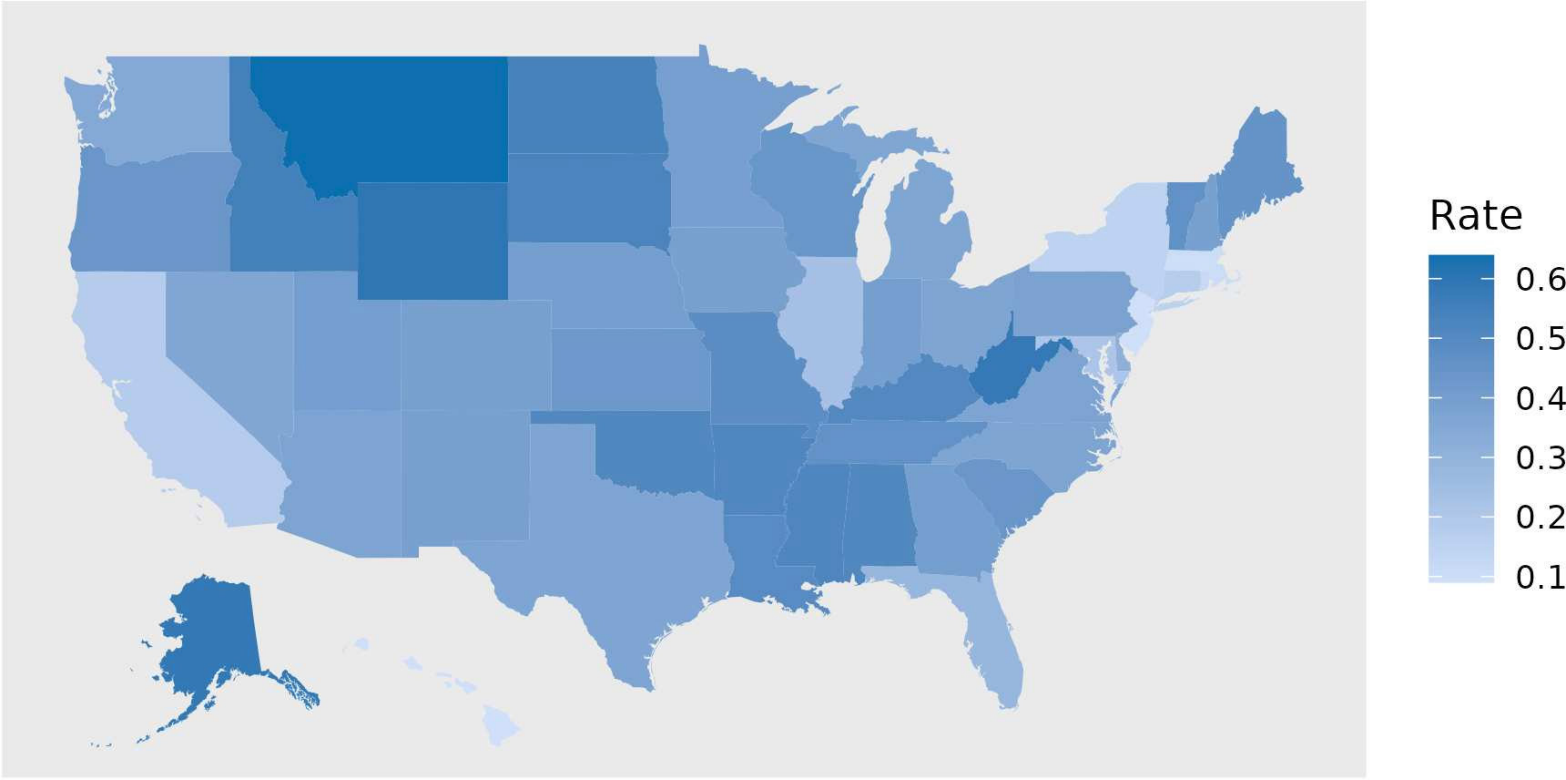
Mean household firearm ownership rates by US state, 2002–2019 (N=50 states).

Annual state household firearm ownership rate estimates (HFR) were drawn from Schell *et al*,^21^ which includes estimates for study years 1990–2016. HFR changes slowly, so we impute rates for the 2017–2019 by carrying forward the 2016 estimates (see figure 1).

**Figure 2:**
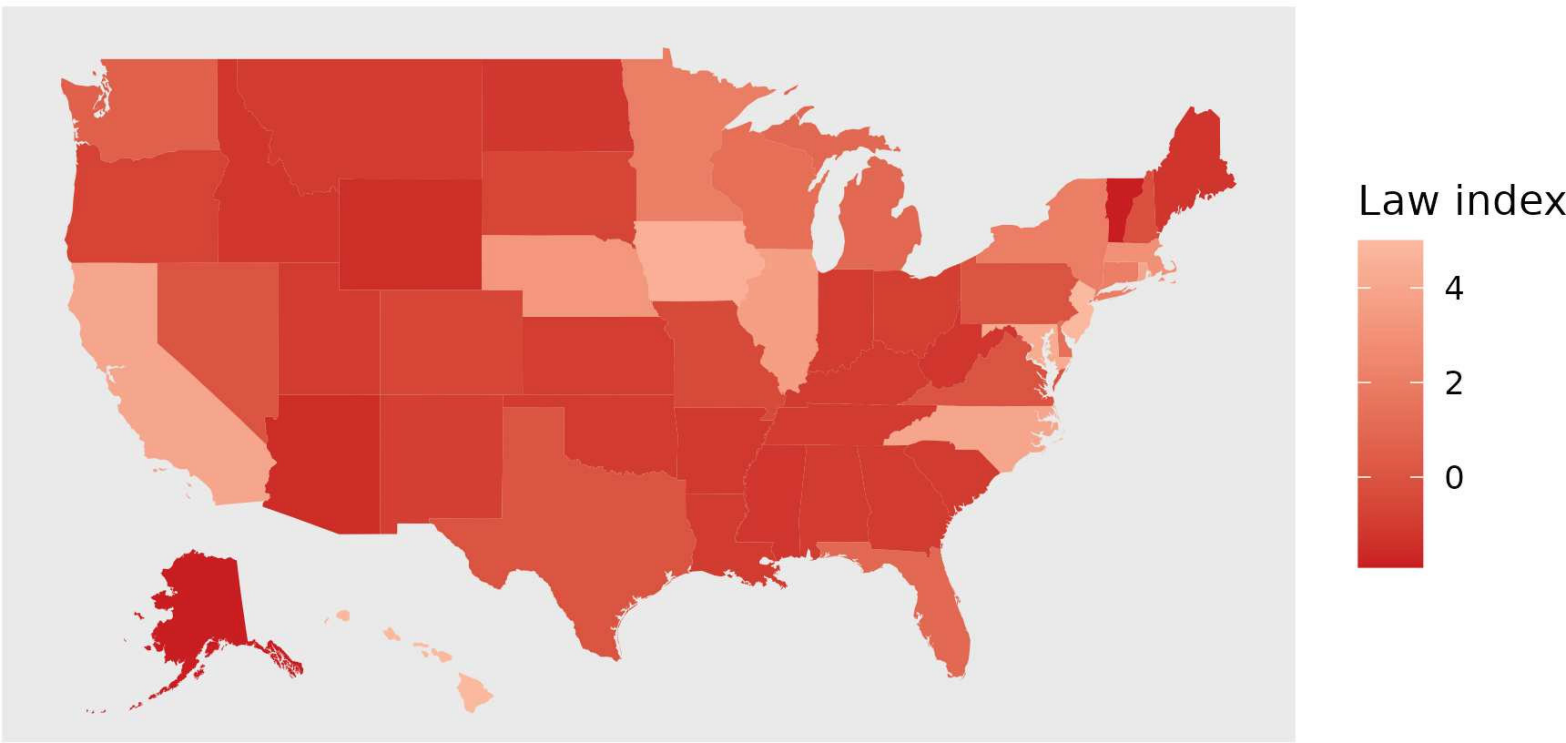
Mean law restrictiveness index score by US state, 2002–2019 (N=50 states).

An index of firearm policy restrictiveness was constructed for each state and 3-year period using data from RAND’s State Firearm Law database.^22^ The index tabulates the presence of seven laws selected because they are designed to reduce suicides (among other intended effects) or because they have been shown to be associated with suicide risk:^14^ (1) background checks on private sales, (2) waiting periods of 24 hours or more to purchase firearms; (3) waiting periods of 7 days or more; (4) child-access prevention (safe storage) laws; (5) ‘shall-issue’ concealed carry laws that prohibit government from exercising discretion in the issuance of permits (reverse coded); (6) permitless carry laws which allow concealed carry without a permit (reverse coded); and (7) laws that require a permit and background check to acquire any firearm (see figure 2).

### Statistical analysis

We modelled state-level suicide rates from 2002 to 2019, where rates were stratified by veteran status (veteran or matched non-veterans) and by lethal means (firearm or non-firearm) within each 3-year period. We used a Bayesian negative binomial model of state suicide deaths with a population offset. Predictors included: veteran status, lethal means, HFR, firearm law index, state and period effects and several covariates, discussed below. Several interaction terms were included to permit HFR, firearm law index and time to have different associations with suicide risk as a function of veteran status and lethal means (see model description in supplemental materials).

Initial model development began with a complex model that included state and period fixed effects, as well as 21 state-level covariates that captured the demographic, socioeconomic and population health characteristics of each state in each period (Table S1). Because controlling for this large set of interrelated predictors may not provide the most accurate model estimates, the model was simplified whenever that change resulted in improvements in an estimate of out-of-sample prediction error, the leave-one-out crossvalidation information criterion (LOOIC).^23^ The final, best-fitting model used random effects for state and period and retained two covariates: percentage of residents who are black and population density. Results from the complex model are included in the supplemental materials. It produced estimates in the same direction and approximate magnitude as the final model, although with wider CIs.

We present results using marginal effects expressed as suicide rates per 100 000 person-years. These effects are derived from the posterior distributions of expected deaths when setting specified predictors to identified values, while maintaining the empirical distribution of other predictors. The reported estimates are the posterior median, and the 95% credible interval (CrI) lower and upper limits are the 2.5th and 97.5th quantiles of the posterior distributions. Because the model includes interactions with time, marginal effects are computed for the most recent period, 2017–2019.

Ethics approval. RAND’s Institutional Review Board approved this research on 9 September 2022.

## Results

The average suicide rate over the full period was 28.2 per 100 000 population (95% confidence interval (CI): 28.0, 28.4) among veterans and 27.5 (95% CI: 27.4, 27.6) among matched non-veterans (here and throughout this discussion, rates are expressed as deaths per 100 000 population). For both groups, most suicides involved a firearm. For veterans, the overall rate was composed of 19.0 (95% CI: 18.9, 19.2) and 9.2 (95% CI: 9.1, 9.3), for firearm and non-firearm suicides, respectively. For matched non-veterans these rates were 17.6 (95% CI: 17.5, 17.7) and 9.9 (95% CI: 9.9, 10.0).

**Figure 3:**
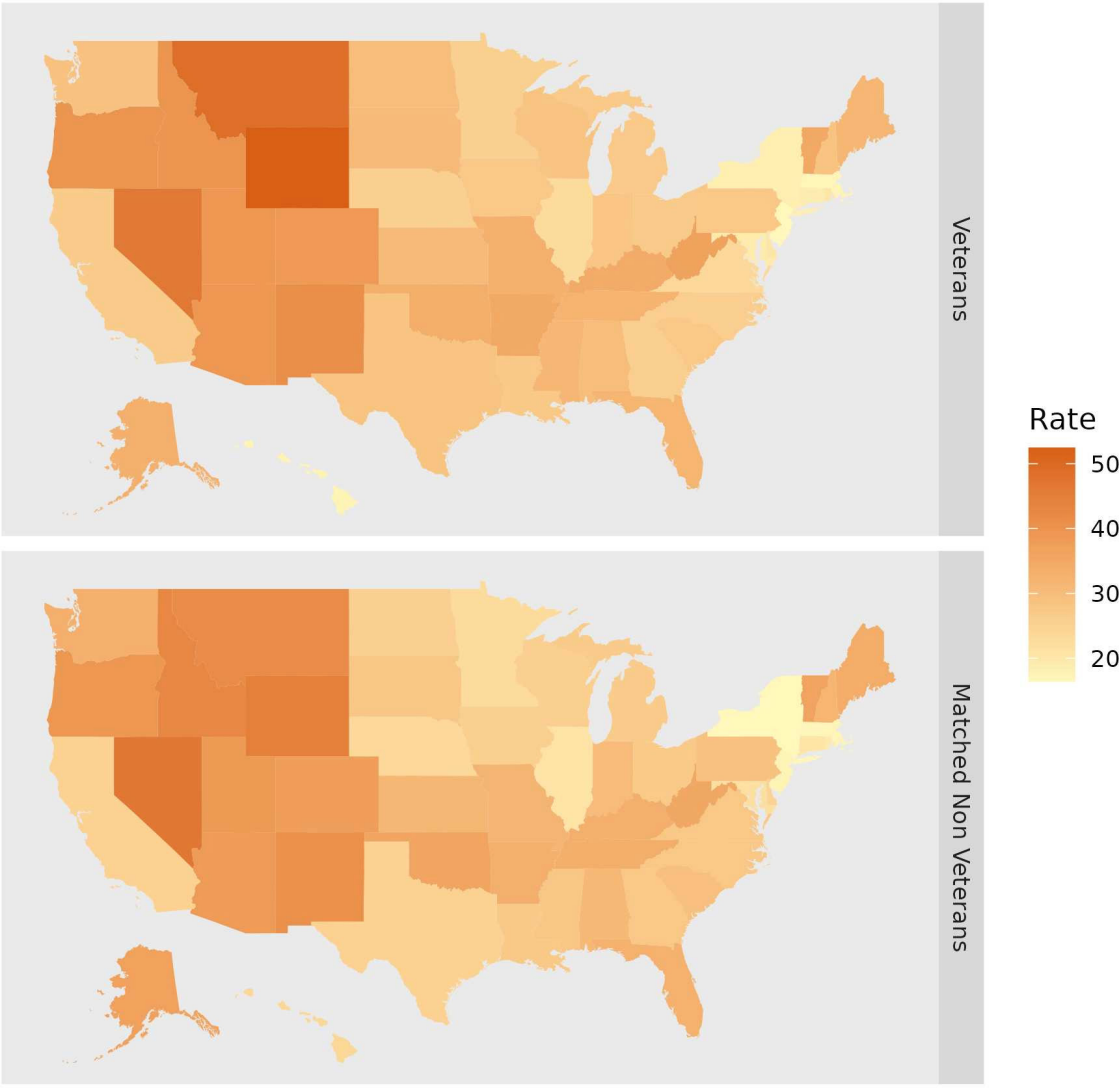
Mean suicide rates by US state and veteran status, 2002–2019 (N=50 states).

Among veterans and matched non-veterans there were large differences across US states in suicide rates, with the maximum average state rate being three times greater than the minimum over the study period. The geographical pattern in suicide rates was similar for the veteran and matched non-veteran populations (figure 3), and the correlation in rates across states between the two groups was high (r=0.94), the substantial variance in suicide risk across states is primarily due to the differences in firearm suicide rates. For veterans and non-veterans the SD across states in firearm suicide rates was three times greater than for non-firearm suicides (veterans’ SDs=7.3 and 2.1, respectively; non-veterans’ SD=7.2 and 2.4). Table 1 provides state suicide rates by veteran status and lethal means (firearm vs non-firearm).

**Table 1:** Suicide rates per 100 000 population over 2002–2019 by US state, veteran status and method of suicide

State	N		Total suicide		Firearm suicide		Non-firearm suicide	
	Veteran	Non-veteran*	Veteran	Non-veteran*	Veteran	Non-veteran*	Veteran	Non-veteran[Table-fn T1_FN2]
Alabama	1 232 667	2 340 530	30.1 (28.9 to 31.4)	31.1 (30.1 to 32.0)	23.9 (22.8 to 25.1)	24.6 (23.7 to 25.4)	6.2 (5.6 to 6.7)	6.5 (6.1 to 6.9)
Alaska	218 333	400 654	32.9 (29.8 to 36.0)	36.7 (34.3 to 39.2)	25.7 (23.0 to 28.5)	25.7 (23.7 to 27.7)	7.2 (5.7 to 8.6)	11.0 (9.7 to 12.4)
Arizona	1 613 667	2 558 370	39.2 (37.9 to 40.4)	38.3 (37.3 to 39.3)	29.0 (27.9 to 30.0)	26.9 (26.0 to 27.7)	10.2 (9.6 to 10.9)	11.5 (10.9 to 12.0)
Arkansas	750 833	1 378 019	34.9 (33.1 to 36.6)	33.5 (32.2 to 34.7)	25.4 (23.9 to 26.8)	25.9 (24.8 to 27.0)	9.5 (8.6 to 10.4)	7.6 (7.0 to 8.2)
California	6 136 333	16 187 315	26.5 (26.0 to 27.0)	25.0 (24.7 to 25.3)	15.9 (15.5 to 16.3)	13.4 (13.2 to 13.7)	10.6 (10.3 to 10.9)	11.6 (11.3 to 11.8)
Colorado	1 261 667	2 280 441	38.4 (37.0 to 39.8)	37.3 (36.3 to 38.3)	24.9 (23.8 to 26.1)	23.2 (22.4 to 24.0)	13.5 (12.7 to 14.3)	14.1 (13.5 to 14.7)
Connecticut	698 167	1 346 120	18.7 (17.4 to 20.1)	20.6 (19.6 to 21.6)	8.8 (7.9 to 9.7)	9.1 (8.5 to 9.8)	9.9 (9.0 to 10.9)	11.5 (10.7 to 12.2)
Delaware	234 500	394 426	20.6 (18.2 to 23.0)	24.8 (22.8 to 26.9)	12.9 (11.1 to 14.8)	13.9 (12.4 to 15.4)	7.7 (6.2 to 9.1)	10.9 (9.6 to 12.2)
Florida	5 080 333	9 542 776	31.9 (31.2 to 32.5)	32.7 (32.2 to 33.2)	21.8 (21.3 to 22.4)	20.1 (19.7 to 20.4)	10.1 (9.7 to 10.4)	12.6 (12.3 to 12.9)
Georgia	2 223 500	4 910 396	26.0 (25.1 to 26.8)	27.6 (27.0 to 28.2)	19.8 (19.0 to 20.5)	20.3 (19.8 to 20.8)	6.2 (5.8 to 6.6)	7.3 (7.0 to 7.6)
Hawaii	360 833	782 736	17.2 (15.5 to 19.0)	23.8 (22.5 to 25.2)	5.7 (4.7 to 6.7)	5.6 (5.0 to 6.3)	11.5 (10.1 to 13.0)	18.2 (17.0 to 19.4)
Idaho	392 500	646 455	39.7 (37.2 to 42.3)	42.8 (40.8 to 44.9)	30.0 (27.8 to 32.2)	31.9 (30.2 to 33.7)	9.8 (8.5 to 11.0)	10.9 (9.8 to 11.9)
Illinois	2 329 667	5 178 346	22.6 (21.8 to 23.4)	21.0 (20.5 to 21.6)	13.4 (12.8 to 14.0)	10.6 (10.2 to 10.9)	9.2 (8.7 to 9.7)	10.5 (10.1 to 10.8)
Indiana	1 476 000	2 833 829	27.8 (26.7 to 28.9)	30.2 (29.4 to 31.1)	19.3 (18.3 to 20.2)	20.4 (19.7 to 21.1)	8.5 (7.9 to 9.2)	9.8 (9.4 to 10.3)
Iowa	723 167	1 315 099	27.7 (26.1 to 29.2)	25.6 (24.4 to 26.7)	17.0 (15.7 to 18.2)	15.5 (14.6 to 16.4)	10.7 (9.7 to 11.7)	10.1 (9.4 to 10.8)
Kansas	688 167	1 273 271	30.5 (28.9 to 32.2)	31.5 (30.2 to 32.8)	20.6 (19.2 to 22.0)	21.6 (20.5 to 22.6)	10.0 (9.0 to 10.9)	9.9 (9.2 to 10.6)
Kentucky	1 007 833	2 131 903	34.2 (32.7 to 35.7)	33.5 (32.5 to 34.5)	25.5 (24.3 to 26.8)	25.5 (24.6 to 26.4)	8.6 (7.9 to 9.4)	8.0 (7.5 to 8.5)
Louisiana	994 667	2 329 063	27.3 (26.0 to 28.6)	27.2 (26.3 to 28.0)	21.2 (20.1 to 22.4)	20.6 (19.8 to 21.3)	6.0 (5.4 to 6.7)	6.6 (6.2 to 7.0)
Maine	397 833	624 969	31.6 (29.3 to 33.8)	33.9 (32.0 to 35.8)	21.3 (19.4 to 23.1)	22.6 (21.1 to 24.2)	10.3 (9.0 to 11.6)	11.3 (10.2 to 12.3)
Maryland	1 349 500	2 865 598	19.3 (18.4 to 20.3)	21.6 (20.9 to 22.3)	12.3 (11.5 to 13.0)	12.6 (12.1 to 13.1)	7.1 (6.5 to 7.6)	9.0 (8.6 to 9.5)
Massachusetts	1 240 667	2 437 486	17.1 (16.1 to 18.0)	17.2 (16.5 to 17.8)	6.6 (6.0 to 7.2)	5.6 (5.2 to 5.9)	10.5 (9.7 to 11.2)	11.6 (11.0 to 12.1)
Michigan	2 163 500	4 223 368	27.0 (26.1 to 27.9)	26.5 (25.9 to 27.2)	17.8 (17.1 to 18.6)	17.2 (16.7 to 17.7)	9.2 (8.7 to 9.7)	9.3 (9.0 to 9.7)
Minnesota	1 165 833	2 008 987	25.4 (24.2 to 26.6)	22.6 (21.8 to 23.5)	15.2 (14.3 to 16.1)	13.7 (13.0 to 14.4)	10.2 (9.5 to 11.0)	8.9 (8.4 to 9.5)
Mississippi	652 333	1 522 566	31.6 (29.8 to 33.3)	27.7 (26.7 to 28.8)	25.6 (24.0 to 27.2)	22.1 (21.1 to 23.0)	6.0 (5.2 to 6.7)	5.7 (5.2 to 6.2)
Missouri	1 522 167	2 530 426	33.2 (32.0 to 34.4)	31.9 (31.0 to 32.8)	24.0 (23.0 to 25.0)	22.8 (22.1 to 23.6)	9.2 (8.6 to 9.9)	9.1 (8.6 to 9.6)
Montana	300 333	466 980	48.9 (45.7 to 52.2)	41.8 (39.4 to 44.2)	36.6 (33.8 to 39.4)	30.0 (28.0 to 32.0)	12.4 (10.8 to 14.0)	11.8 (10.5 to 13.0)
Nebraska	442 500	762 408	25.4 (23.5 to 27.3)	23.3 (21.9 to 24.7)	17.4 (15.8 to 19.0)	15.1 (14.0 to 16.3)	8.0 (6.9 to 9.1)	8.2 (7.4 to 9.1)
Nevada	696 167	1 164 937	46.3 (44.2 to 48.4)	46.5 (44.9 to 48.1)	31.0 (29.3 to 32.7)	31.0 (29.7 to 32.3)	15.3 (14.1 to 16.5)	15.5 (14.6 to 16.4)
New Hampshire	355 833	533 053	28.4 (26.2 to 30.7)	31.8 (29.9 to 33.8)	18.3 (16.5 to 20.1)	18.7 (17.2 to 20.2)	10.2 (8.8 to 11.5)	13.2 (11.9 to 14.4)
New Jersey	1 416 667	3 169 054	16.4 (15.6 to 17.3)	17.3 (16.7 to 17.9)	8.0 (7.4 to 8.6)	6.6 (6.3 to 7.0)	8.4 (7.8 to 9.1)	10.6 (10.1 to 11.1)
New Mexico	519 333	924 103	41.1 (38.9 to 43.4)	40.9 (39.2 to 42.5)	28.8 (26.9 to 30.7)	28.0 (26.7 to 29.4)	12.4 (11.1 to 13.6)	12.8 (11.9 to 13.7)
New York	2 988 833	8 186 276	18.3 (17.7 to 18.9)	16.4 (16.1 to 16.8)	9.7 (9.2 to 10.1)	6.4 (6.2 to 6.7)	8.6 (8.2 to 9.0)	10.0 (9.7 to 10.3)
North Carolina	2 301 500	4 877 939	25.9 (25.1 to 26.8)	27.6 (27.0 to 28.2)	18.7 (18.0 to 19.4)	19.8 (19.2 to 20.3)	7.2 (6.7 to 7.6)	7.8 (7.5 to 8.2)
North Dakota	178 000	386 620	30.1 (26.9 to 33.4)	25.7 (23.7 to 27.8)	20.3 (17.6 to 23.0)	16.7 (15.1 to 18.4)	9.8 (8.0 to 11.7)	9.0 (7.8 to 10.2)
Ohio	2 773 667	5 089 117	26.8 (26.0 to 27.6)	27.1 (26.5 to 27.7)	18.0 (17.3 to 18.6)	17.5 (17.1 to 18.0)	8.8 (8.4 to 9.3)	9.6 (9.2 to 9.9)
Oklahoma	997 833	1 689 913	33.6 (32.1 to 35.0)	35.8 (34.6 to 37.0)	24.3 (23.1 to 25.6)	26.2 (25.2 to 27.2)	9.3 (8.5 to 10.0)	9.6 (9.0 to 10.2)
Oregon	1 021 833	1 615 474	39.6 (38.0 to 41.2)	39.1 (37.8 to 40.3)	27.7 (26.3 to 29.0)	26.2 (25.2 to 27.3)	11.9 (11.1 to 12.8)	12.9 (12.1 to 13.6)
Pennsylvania	2 992 667	4 887 119	27.5 (26.7 to 28.3)	28.7 (28.0 to 29.3)	18.2 (17.6 to 18.8)	18.4 (17.9 to 18.9)	9.3 (8.9 to 9.7)	10.2 (9.8 to 10.6)
Rhode Island	230 500	407 748	20.6 (18.2 to 23.0)	19.1 (17.4 to 20.9)	9.0 (7.5 to 10.6)	5.9 (5.0 to 6.9)	11.6 (9.8 to 13.4)	13.2 (11.8 to 14.6)
South Carolina	1 237 000	2 245 273	27.6 (26.4 to 28.8)	29.4 (28.5 to 30.3)	20.4 (19.4 to 21.5)	22.0 (21.2 to 22.8)	7.2 (6.5 to 7.8)	7.4 (6.9 to 7.8)
South Dakota	223 667	393 370	30.6 (27.7 to 33.6)	27.7 (25.6 to 29.8)	20.0 (17.6 to 22.4)	18.6 (16.9 to 20.4)	10.6 (8.8 to 12.3)	9.1 (7.9 to 10.3)
Tennessee	1 529 000	3 135 056	32.3 (31.1 to 33.5)	33.7 (32.9 to 34.5)	24.1 (23.1 to 25.1)	25.4 (24.7 to 26.1)	8.2 (7.6 to 8.8)	8.3 (7.9 to 8.7)
Texas	5 022 667	11 919 450	28.4 (27.8 to 29.0)	24.9 (24.6 to 25.3)	20.7 (20.2 to 21.2)	17.4 (17.1 to 17.7)	7.8 (7.4 to 8.1)	7.5 (7.3 to 7.8)
Utah	446 833	872 824	39.3 (36.9 to 41.7)	38.6 (36.9 to 40.3)	26.1 (24.2 to 28.0)	25.8 (24.4 to 27.2)	13.2 (11.8 to 14.6)	12.8 (11.8 to 13.7)
Vermont	145 667	289 160	35.0 (31.1 to 38.9)	36.1 (33.3 to 39.0)	23.5 (20.2 to 26.7)	25.3 (22.9 to 27.7)	11.6 (9.3 to 13.8)	10.8 (9.3 to 12.4)
Virginia	2 301 167	4 454 124	23.3 (22.5 to 24.1)	27.4 (26.8 to 28.0)	16.3 (15.6 to 17.0)	18.8 (18.2 to 19.3)	7.0 (6.6 to 7.5)	8.6 (8.3 to 9.0)
Washington	1 850 000	3 021 506	28.6 (27.6 to 29.6)	33.3 (32.5 to 34.1)	18.2 (17.4 to 19.0)	21.0 (20.3 to 21.7)	10.4 (9.8 to 11.0)	12.3 (11.8 to 12.8)
West Virginia	494 500	883 590	36.4 (34.3 to 38.6)	35.2 (33.6 to 36.8)	29.0 (27.0 to 30.9)	27.8 (26.3 to 29.2)	7.5 (6.5 to 8.5)	7.5 (6.7 to 8.2)
Wisconsin	1 289 833	2 392 773	28.4 (27.3 to 29.6)	25.7 (24.9 to 26.5)	17.7 (16.8 to 18.7)	15.6 (15.0 to 16.3)	10.7 (10.0 to 11.5)	10.1 (9.6 to 10.6)
Wyoming	146 167	277 438	52.5 (47.7 to 57.2)	44.7 (41.5 to 47.9)	40.0 (35.8 to 44.2)	34.2 (31.4 to 37.0)	12.4 (10.1 to 14.8)	10.5 (9.0 to 12.1)

Note: 95% CIs are in parenthesis. Population sizes (N) are average populations across the 3-year intervals.

* Non-veterans are weighted to match to the veteran population on age and gender and are not representative of the broader state populations. Non-veteran N is the effective sample size of the weighted non-veteran population.

Predicted values from the model of state suicide rates were highly associated with the actual suicide rates, R^2^=0.90 suggesting a good model fit. Additional information about model fit and posterior distributions of all parameters is contained in the supplemental materials.

To evaluate the modelled association of HFR with suicide rates, we estimated marginal effects in the 2017–2019 period, comparing predicted suicide rates when setting states to a low (35.3%) versus a high HFR (52.3%) value. These correspond to the 25th and 75% percentiles of state HFR in 2017–2019. Among veterans, the high HFR value was associated with a suicide rate 4.35 per 100 000 (95% CrI: 1.90, 7.14) higher than with a low HFR. For matched non-veterans it was also associated with higher rates (3.31; 95% CrI: 1.11, 5.77), and there was little evidence that the association of HFR and suicide rates is meaningfully greater among veterans than non-veterans (difference of 1.03; 95% CrI: −0.23, 2.43).

Decomposing the association of HFR and suicide rates into the firearm and non-firearm suicide rates reveals that this positive association is entirely explained by the positive association of HFR with firearm suicides. The association with firearm suicide was positive for veterans (5.85 deaths per 100 000; 95% CrI: 3.92, 8.01) and matched non-veterans (5.77; 95% CrI: 4.06, 7.66). In contrast, the association of HFR with non-firearm suicide rates was negative for veterans (−1.49 95% CrI: −2.18 to –0.73) and non-veterans (−2.46; 95% CrI: −3.05 to –1.78). There was little evidence that the association of HFR and firearm suicide rates differed between veterans and non-veterans (difference of 0.08 deaths per 100 000; 95% CrI: −1.16, 1.37). Although the negative association of HFR with non-firearm suicides was stronger for non-veterans than veterans (difference of 0.96; 95% CrI: 0.47, 1.43), or both groups the HFR’s small negative association with non-firearm suicide was fully offset by the large positive association with firearm suicides.

Marginal effects for state firearm law restrictiveness on suicide rates were computed by contrasting 25th and 75th percentile values for state law restrictiveness in 2017–2019, which corresponds to a difference of three laws. Greater law restrictiveness was associated with fewer suicides among both veterans (−2.49 per 100 000; 95% CrI: −4.64 to –0.21), and non-veterans (−3.19; 95% CrI: −5.22 to –1.16). There was little evidence that these two effects differed in strength (difference in estimates: 0.70; 95% CrI: −0.67, 2.03).

The association between firearm law restrictiveness and overall suicide rates was almost entirely attributable to its association with firearm suicides. Expected suicide rates associated with low versus high law restrictiveness were larger for firearm suicides than non-firearm suicides over the two populations (difference of 4.7; 95% CrI: 3.0 to 6.4). Greater law restrictiveness was associated with fewer firearm suicides among both veterans (−2.44 deaths per 100 000; 95% CrI: −4.00 to –0.78), and matched non-veterans (−2.68; 95% CrI: −4.02 to –1.36). In contrast, law restrictiveness was not reliably associated with non-firearm suicide rates for either veterans (−0.06; 95% CrI: −0.87, 0.77) or non-veterans (−0.50; 95% CrI: −1.35, 0.35). There was little evidence that these associations with law restrictiveness differed between veterans and matched non-veterans for either firearm suicide rates (difference of 0.26; 95% CrI: −0.93, 1.40), or non-firearm suicide rates (difference of 0.45; 95% CrI: −0.26, 1.13).

## Discussion

Veteran suicide rates, particularly their firearm suicide rates, vary dramatically across states. This state variation in risk was associated with HFR and with the restrictiveness of state firearm laws. The association of both state characteristics with suicide rates was highly specific to the subset of suicides completed with a firearm. The effect size for the association between suicide rates and HFR is substantial. With a 17% point increase in HFR, the expected veteran suicide rate shifts 14% (inter-quartile range (IRR)=1.14; 95% CrI: 1.06, 1.22), which corresponds to 870 more veteran deaths nationally in 2021.^1^

These state firearm characteristics had similar associations with suicide risk for veterans and matched non-veterans, although the positive association between HFR and non-firearm suicides was slightly larger for non-veterans than veterans. These similarities occur despite veterans having a unique relationship to firearms that differs from their non-veteran neighbours, due to their extensive firearm training, military experience and higher average rates of firearm ownership.^3^

Our estimates are correlational, and we do not interpret them as causal effect estimates. This reflects the fact that most interstate variation in gun ownership and firearm laws predates the beginning of the available VA suicide data. Thus, the data do not support a stronger analytical approach that might identify causal effects. Restricting the analysis so that modelled effects are identified solely through the limited changes in state firearm ownership and in firearm policies during the study period yields imprecise effect estimates (Tables S4-S5).

There are, however, four reasons why the current results should be seen as supportive of theories that hypothesise a causal link between the state firearm environment and suicide risk: (1) The estimates controlled for a range of state characteristics thought to be confounds for firearm environment variables (The estimated effects of state firearm ownership and firearm law restrictiveness are from the same model where each controls for the effect of the other. These constructs are plausibly mutually endogenous, which would make the modelled effects conservative estimates of the underlying causal effects for one or both variables.) as well as state random intercepts which can capture differences in risk between states caused by state characteristics that are not explicitly included in the model. (2) Associations were found prospectively within a longitudinal model that covers an 18-year period. (3) The associations of the state firearm environment variables were highly specific to firearm suicide rates; to the extent that the reported associations represent some omitted confounding variable, that variable must have sharply differentiated effects on firearm and non-firearm suicide rates—without itself being a state firearm characteristic. And (4) some of the estimated associations are large. For these association to be fully explained by omitted confounds requires that the current model failed to capture some especially strong predictors of firearm suicide risk that vary dramatically across states.

Finally, the results of this study are consistent with other research suggesting that firearm ownership and state-level policies affect suicide risk. For instance, other research shows that the suicide risk increases substantially in the period shortly after an individual purchases a firearm,^2^ and is substantially higher than for similar individuals who did not purchase firearms.^24^ Thus, while one should not interpret our results as suggesting that a shift in HFR from 35% to 52% has the causal effect of increasing veteran firearm suicides by 5.8 per 100 000, our findings are consistent with the theory that state firearm policies and firearm ownership affect suicide risk.

In addition to the limitations inherent in interpreting associations in observational data, the data themselves present limitations. The VA does not share microdata on veteran suicide. As such, our construction of a matched comparison sample of non-veterans required estimating how many veteran decedents to remove from the general population suicide totals within the cells of a five-way table of decedent characteristics based on the publicly released three-way tables of veteran suicide decedents (see supplemental materials). Despite this imprecision, the matched comparison sample appeared to have suicide risk that was highly comparable to veterans’ risks. While the counts of veteran suicides we used are the most complete and carefully constructed data available, they are known to undercount suicides among veterans who separated from the military prior to 1974.^20^ This is likely to result in a slight underestimate of veteran suicide rates for the oldest cohort of veterans, particularly in the earlier periods of this study.

## Conclusions

One of the five priority goals for reducing military and veteran suicide announced by the White House was addressing upstream risk and protective factors, such as economic well-being, access to community resources and educational and vocational opportunities all of which have been linked to suicide risk.^25^ This study identifies two candidate social determinants of suicide that have strong associations with suicide risk among veterans and non-veterans, and which states have considerable control over: the restrictiveness of state firearm policies and state firearm ownership rates. These factors can help identify groups of veterans who are at unusually high risk for suicide to better target existing prevention programmes or resources. In addition, the results suggest that changes to state firearm laws and policies should be investigated as a possibly cost-effective primary prevention strategy for reducing suicide rates among veterans and non-veterans.

## Supplementary material

10.1136/ip-2023-045211 online supplemental file 1

## Data Availability

Data are available upon reasonable request.

## References

[1] Office of Mental Health and Suicide Prevention (2023). 2023 national veteran suicide prevention annual report.

[2] Studdert DM, Zhang Y, Swanson SA (2020). Handgun Ownership and Suicide in California. N Engl J Med.

[3] Cleveland EC, Azrael D, Simonetti JA (2017). Firearm ownership among American veterans: findings from the 2015 National Firearm Survey. Inj Epidemiol.

[4] Pompili M, Gonda X, Serafini G (2013). Epidemiology of suicide in bipolar disorders: a systematic review of the literature. Bipolar Disord.

[5] Kimerling R, Makin-Byrd K, Louzon S (2016). Military Sexual Trauma and Suicide Mortality. Am J Prev Med.

[6] Brenner LA, Ignacio RV, Blow FC (2011). Suicide and traumatic brain injury among individuals seeking Veterans Health Administration services. J Head Trauma Rehabil.

[7] Kang HK, Bullman TA, Smolenski DJ (2015). Suicide risk among 1.3 million veterans who were on active duty during the Iraq and Afghanistan wars. Ann Epidemiol.

[8] Sokol Y, Gromatsky M, Edwards ER (2021). The deadly gap: Understanding suicide among veterans transitioning out of the military. Psychiatry Res.

[9] Ilgen MA, Bohnert ASB, Ignacio RV (2010). Psychiatric diagnoses and risk of suicide in veterans. Arch Gen Psychiatry.

[10] (2023). 1999-2020: underlying cause of death by bridged race categories. National Center for Health Statistics.

[11] Stack S (2021). Contributing factors to suicide: Political, social, cultural and economic. Prev Med.

[12] Flavin P, Radcliff B (2009). Public Policies and Suicide Rates in the American States. Soc Indic Res.

[13] Ahern J (2020). Minimum wage policy protects against suicide in the USA. J Epidemiol Community Health.

[14] Smart R, Morral AR, Ramchand R (2023). The Science of Gun Policy: A Critical Synthesis of Research Evidence of the Effects of Gun Policies in the United States.

[15] Hawton K, Casañas I Comabella C, Haw C (2013). Risk factors for suicide in individuals with depression: a systematic review. J Affect Disord.

[16] Miller M, Azrael D, Barber C (2012). Suicide mortality in the United States: the importance of attending to method in understanding population-level disparities in the burden of suicide. Annu Rev Public Health.

[17] Bailey AK (2011). Race, Place, and Veteran Status: Migration among Black and White Men, 1940–2000. Popul Res Policy Rev.

[18] Office of Mental Health and Suicide Prevention (2023). 2023 national veteran suicide prevention annual report: methods summary.

[19] Belsher BE, Smolenski DJ, Pruitt LD (2019). Prediction Models for Suicide Attempts and Deaths: A Systematic Review and Simulation. JAMA Psychiatry.

[20] Hoffmire CA, Barth SK, Bossarte RM (2020). Reevaluating Suicide Mortality for Veterans With Data From the VA-DoD Mortality Data Repository, 2000-2010. Psychiatr Serv.

[21] Schell T, Peterson S, Vegetabile B (2020). State-Level Estimates of Household Firearm Ownership.

[22] Cherney S, Morral AR, Schell TL (2022). Development of the RAND State Firearm Law Database and Supporting Materials.

[23] Vehtari A, Gelman A, Gabry J (2017). Practical Bayesian model evaluation using leave-one-out cross-validation and WAIC. Stat Comput.

[24] Schleimer JP, Kagawa RMC, Laqueur HS (2021). Handgun purchasing characteristics and firearm suicide risk: a nested case-control study. Inj Epidemiol.

[25] House W (2021). Reducing Military and Veteran Suicide: Advancing a Comprehensive, Cross-Sector, Evidence-Informed Public Health Strategy.

