# Supplementary material for: Association of veteran suicide risk with state-level firearm ownership rates and firearm laws in the USA
Source: Inj Prev. 2024 Sep 30;32(1):e045211. doi: 10.1136/ip-2023-045211 (PMC12911575; doi:10.1136/ip-2023-045211)
Supplement: online supplemental file 1 [file ip-32-1-s001.docx]

**Supplemental Materials:**

**The association of veteran suicide risk with state-level firearm ownership rates and firearm laws in the United States.**

Andrew R Morral, Terry L Schell, Adam Scherling

**Methods**

**Nonveteran comparison sample**. Data on suicide rates in the total population comes from CDC Wonder.(1) To construct the matched comparison sample of nonveterans veteran decedents were first removed from the full population suicide totals. This was done for each cell (N=3600) of a five-way table defined by the combination of six time periods, fifty states, two genders, three age groups (25-44, 45-64, and 65 and older), and two methods of suicide (firearm, nonfirearm).

For privacy reasons, the VA will not provide exact counts of veteran suicides for the individual cells of this data table. However, we can closely approximate those counts using data that is publicly available. Specifically, the VA has publicly released (via their annual report, or via https://www.mentalhealth.va.gov/suicide_prevention/data.asp), or have provided directly to RAND, tables that represent the key margins of the full table of interest. These include the following three-way tables of suicide counts: time period by state by sex, time period by method by sex, time period by state by method, and time period by method by age. We estimated the suicide counts in the cells of the full five-way table of interest ensuring that all such three-way tables were reproduced. Specifically, the individual cells of the table were imputed so that they summed to the corresponding cell values in each of the available three-way tables but were otherwise assumed to be independent. For example, the number of female suicide decedents between the ages of 18 and 45 in period 1 in Alabama who used a firearm is not available in any provided table. However, we know (a) the number of female decedents in Alabama in period 1, (b) the number of decedents age 18-45 in period 1 in Alabama, (c) the number of firearm decedents in Alabama in period 1, (d) the association between sex and the likelihood of using a firearm in suicide in period 1 across all states. One can then estimate female firearm suicide decedents in period 1 in Alabama that is consistent with those three pieces of information.

Although cell values in the 5-way table are imputed in this way, imprecision in these imputations are likely to have minimal effects for the overall analysis. This table determines the number of veteran decedents who are subtracted from the total population suicide decedents so that we can create a nonveteran suicide rate. However, the proportion of all suicide decedents in the general population who are veterans is just 17 percent over the study period, and so the final suicide rate among nonveterans is expected to be relatively insensitive to exactly how the veterans’ deaths are allocated – the suicide rate for age and gender matched nonveterans is always going to be very close to the suicide rate for the age and gender matched total population regardless of how we allocate the veteran suicide decedents. In addition, we know our allocation is approximately correct because it reproduces all three-way contingency tables discussed above. Finally, these estimated counts only affect suicide rate estimates for the matched nonveteran comparison group. The suicide rates for the veterans in our analyses do not depend in any way on the estimated cell counts in this table because the primary analysis is not stratified by age and gender (only time by state and by method, which is a table provided by the VA); the allocation only affects suicide rates of the matched nonveteran group.

Once estimated counts of veterans’ suicides of each type are subtracted from the population total suicides to create a count of nonveteran suicides, we also subtract the total veteran population sizes (provided by the VA using the VETPOP model) from the population sizes provided in CDC wonder to get a nonveteran population estimate so that nonveteran suicide rates can be computed.

We then use balancing weights to match the population age and sex distribution of the nonveteran to the distribution of veterans within each state and time period. This indirect standardization allows for comparisons of suicide rates among veterans and a demographically similar nonveteran comparison group. Weights are only applied to the nonveteran group in the analysis. Weighting is done using six strata defined by the two genders by three age groups within each state and period. The older-male weighting stratum is the one in which veterans are most overrepresented relative to the general population. It is treated as the reference group for the development of initial balancing weights. The weights for all strata within a state and time period are calculated as

$$w_{i}=v_{i}/k_{i}$$

Where for each weighting stratum *i* (young males; young females; middle aged males; middle aged females, older females), *v_i_* is the ratio of the veteran population size in stratum *i* relative to the population size of veterans in the reference stratum (older male), and *k_i_* the ratio of the nonveteran population size in stratum *i* relative to the population size of nonveterans in the reference stratum.

We create weighted nonveteran suicide rates for both firearm and nonfirearm suicide within each period and state. However, our primary analysis considers the population sizes on which each rate was computed in estimating the uncertainty of model-based estimates. This quantity depends on the scale used for the weights, even though the nonveteran suicide rates themselves do not depend on the scale used for the weights., Our final weights are scaled so that the sum of the nonveteran population across the weighting strata equals the effective sample size of the weighted suicide rate estimate. This was done using Kish’s formula for effective sample size.(2)

**Statistical model**. We used a Bayesian negative binomial model of state suicide deaths, *D*, at time *t* and in state *s*, as a function of a logged population offset (*P*), an intercept (*a*), random effects for each state (*s*) and three-year period (*t*), fixed effects for firearm method (*F*), veteran status (*V*), household firearm ownership (*H*), the firearm law index (*I*), and the standardized demographic and economic covariates (***X***), see Table S1.

The model allows for a range of interactions to account for the possibility that the two state firearm characteristics of interest have different associations with suicide risk as a function of veteran status and firearm vs nonfirearm methods. For each firearm characteristic we include this three-way interaction as well as both two-way interactions. In addition to these planned interactions, during model development we identified several other interactions whose inclusions improved model fit as assessed with LOOIC.(3) These interactions suggest that suicide rates have been increasing faster over time for veterans than for the matched nonveterans, and that this increase was larger for firearm than nonfirearm suicides. These exploratory interactions were fit by allowing one interaction between veteran status and a linear function of time (T), and a second interaction that allows that linear time interaction to differ for firearm suicides. As such, the final model is described by:

$$D_{st} \sim Negative Binomial(\gamma_{st}, \varphi)$$

$${log(\gamma}_{st})=\log\left( P \right)+\alpha+\boldsymbol{\lambda}_{s}+\boldsymbol{\gamma}_{t}+{\alpha F}_{st}+{\beta V}_{st}+{\delta H}_{st}+\boldsymbol{\omega X}_{st}+{\rho V}_{st}F_{st}+\zeta T_{st}V_{st}+\eta H_{st}F_{st}+ \psi H_{st}V_{st}+{{\theta I}_{st}F_{st}+\pi I_{st}F_{st}+\varrho I_{st}V_{st}+\Delta{I_{st}V}_{st}F_{st}+\partial H}_{st}V_{st}F_{st}+\tau T_{st}V_{st}F_{st}$$

$$\boldsymbol{\lambda}_{s}\sim Normal\left( 0,\epsilon\right)$$

$$\boldsymbol{\gamma}_{t}\sim Normal(0,\vartheta)$$

$$\epsilon\sim Cauchy(0,1)$$

$$\vartheta\sim Cauchy(0,1)$$

$$1/\varphi\sim Exponential\left( 3 \right).$$

Priors for all parameters were weakly informative. For most coefficients they were normal(0,1), with the exception of the intercept, normal(2,2), the two random effect standard deviations, $\epsilon$ and $\vartheta$ and the overdispersion parameter, $\varphi.$

**Measurement error in HFR**. Although all predictors in the model are measured with error, in the case of HFR, which are model-based estimates of household firearm ownership, we have information about measurement error. We have opted not to adjust model coefficients for measurement error because 1) the omission of measurement error information is conservative: disattenuating for measurement error would increase the magnitude of modeled associations between HFR and suicide risks; 2) such an adjustment could only be made for HFR even though we have no indication that this variable has more error than other predictors, which may result in greater underestimation of the effect sizes for those predictors (4), and 3) the estimated measurement error in HFR is a small fraction of the variance of this measure. The standard deviation in our 3-year HFR estimates across states and years is 13.5 percentage points.  The average standard error of those modelled estimates is 1.8 percentage points.  This implies that less than 2% of the variance in the HFR measure is measurement error.  That is, the variance of the measure (.135^2) divided by the estimated error variance (.018^2) is just .017.

**Supplemental Results**

**Model fit**. As part of model development, we explored more and less complex models and selected the final model as the one that minimized cross-validated error of prediction as assessed with LOOIC.(3) The final model used random effects for states and periods as well as two covariates (Percent of residents who were Black and Population Density), and had a LOOIC of 11413.9. In comparison, the more complex model with fixed state effects and 21 covariates had LOOIC of 11444.3. see Table S.2 for a comparison of marginal effects between the final and saturated models. The model was well-estimated using rstan 2.32.2. All pareto k diagnostics were less than 0.5, indicating good model fit.(4) Rhat values were all less than 1.01, with no divergent transitions across 4000 post-warmup iterations indicating good model convergence. The effective sample size for all model parameters was greater than 700, indicating adequate tail densities for estimating credible intervals. The effective number of parameters in the final model was 68.7, which is less than the nominal number of modeled parameters (75), due to the hierarchical structure of the state and time effects. Given that the model was estimated on 1200 data points, 68.7 effective parameters pose minimal threat of model overfit.

**Model coefficients**. Table S3 provides a description of the posterior distributions of all model parameters. The article is focused primarily on the marginal effects of state gun ownership rates and firearm law restrictiveness, however estimated parameters for the percentage of the state population that is Black and the state’s population density – the two covariates to remain in the model after pruning – are shown in the table, as are individual state and period effects. Both the proportion of residents who are Black, and population density are negatively associated with state suicide rates, conditional on the other predictors in the model.

State random effects also suggest that some states have substantially higher or lower rates of suicide among veterans and matched nonveterans after accounting for other model covariates. Specifically, Arizona, California, Colorado, Florida, Maryland, Nevada, New Mexico, Oregon, Utah and Wyoming are all associated with higher conditional risk of suicide, while Alaska, Hawaii, Massachusetts, Minnesota, Nebraska, New York, North Dakota, South Dakota, and Texas are associated with lower conditional suicide risk.

**Model results restricting law effect identification to changes over the period**. The associations of suicide with state law restrictiveness reported in the paper identify law associations using both changes to law restrictiveness during the period and differences between state law restrictiveness that predate the period of study. To examine these associations in a way that is insensitive to pre-existing differences in state laws, we reran the model using state law restrictiveness scores for each period that are differenced with state law restrictiveness scores in the first period. Thus, in the first period, each state has a state law restrictiveness of zero, and subsequent restrictiveness scores represent changes in law restrictiveness from the initial period. Tables S4 and S5 highlight key law associations under the model presented in the paper vs the model excluding preexisting state law differences. In general, associations between more restrictive gun laws and veteran suicides have similar point estimates, but the estimates are far more uncertain for both veterans and nonveterans. The standard errors of the estimates are frequently more than twice as large for those that restrict identifying variation to solely the changes in state laws within the study period. Table S5 contains estimates for the effect of HFR on suicide excluding the pre-period differences in firearm ownership rates from the identifying variation. Similar to the estimates of the law effects, this estimation approach results in a sufficiently high level of uncertainty that it is not clear what should be concluded from the estimates. Our view is that the available veteran suicide data, which begins in 2001, do not support effect estimates for these variables that exclude pre-period differences between states from the identification. This is because the changes in state firearm laws and state levels of firearm ownership since 2001 are small relative to the difference between states in 2001. In addition, we believe it is useful to document that states with high firearm ownership and permissive firearm laws in 2001 have unusually high risk for firearm suicide almost 20 years later.

**Supplemental References**

1. 1999-2020: Underlying Cause of Death by Bridged Race Categories [Internet]. National Center for Health Statistics. 2023. Available from: <https://wonder.cdc.gov/Deaths-by-Underlying-Cause.html>.

2. Kish L. Survey Sampling. New York: John Wiley & Sons, Inc.; 1965.

3. Vehtari A, Gelman A, Gabry J. Practical Bayesian model evaluation using leave-one-out cross-validation and WAIC. Statistics and computing. 2017;27:1413-32.

4. Vehtari A, Simpson D, Gelman A, Yao Y, Gabry J. Pareto smoothed importance sampling. arXiv preprint arXiv:150702646. 2015.

5. WID.World. World Inequality Database 2023. Available from: <https://wid.world>.

6. National Conference on State Legislators. State Partisan Composition 2023. Available from: <https://www.ncsl.org/about-state-legislatures/state-partisan-composition>.

7. UI Replacement Rates Report [Internet]. 2024. Available from: <https://oui.doleta.gov/unemploy/ui_replacement_rates.asp>.

8. Slater ME, Alpert HR. Surveillance Report #119: Apparent per capita alcohol consumption: National, state, and regional trends, 1977–2020. Bethesda, MD: NIAAA; 2022.

9. Ruggles S, Flood S, Sobek M, Brockman D, Grace Cooper, Richards S, et al. IPUMS USA: Version 13.0 [dataset]. In: IPUMS, editor. Minneapolis, MN2023.

**Supplemental Tables**

**Table S1. State characteristics included as covariates with sources**

| **State Characteristic** | **Source** |
| --- | --- |
| **Demographics** |  |
| Percent Black | US Census Bureau |
| Percent Hispanic | US Census Bureau |
| Percent Age 15-29 | US Census Bureau |
| Percent foreign born | CPS ASEC |
| Percent divorced, separated, or widowed | CPS ASEC |
| Percent urban households | CPS ASEC |
| Population density | US Census Bureau |
|  |  |
| **Socioeconomic conditions** |  |
| Poverty Rate | US Census Bureau |
| Unemployment rate | U.S. Department of Labor, BLS |
| Decile income share | World Inequality Database(5) |
| Average income (inflation adjusted) | Bureau of Economic Analysis |
| Percent without a high-school diploma | CPS ASEC |
| GOP control | National Conference of State Legislatures(6) |
| Unemployment insurance replacement rates | US Dept. of Labor(7) |
|  |  |
| **Population Health** |  |
| Health insurance coverage rate | US Census Bureau |
| Physician population rate | CPS ASEC |
| Therapist and psychologist pop. rate | CPS ASEC |
| Current smoker population rate | BRFSS |
| Per capita alcohol consumption | Slater and Alpert (2023)(8) |
| Adequate sleep population rate | BRFSS |
| Physically active population rate | BRFSS |

Notes: CPS ASEC (Current Population Survey, March Annual Social and Economic Supplement) values calculated using IPUMS data (Ruggles et. al., 2023).(9) *Decile income share* is proportion of state’s personal income associated with the top 10 percent of the income distribution. *GOP control* is the proportion of legislative veto points (typically house, senate, governor) controlled by Republicans using data from the National Conference of State Legislatures. In Nebraska’s nonpartisan legislature, GOP control is determined by a majority of unicameral members with Republican affiliations. *Unemployment insurance replacement rate* is an estimate of the proportion of individuals’ expected salary provided through state unemployment insurance. *Physician population rate* is the proportion of the population with an occupation of physician or surgeon. *Therapist and psychologist population rate* is the proportion of the population with an occupation of psychologist or “therapist, other”, which excludes physical therapists and occupational therapists, but includes psychotherapists and other mental health providers.

**Table S2. Marginal effects of HFR and law restrictiveness on suicide risk per 100,000 for a complex model and the best-fitting model; United States, 2017-2019.**

| **Effect** | **Veterans**  **Median (95% CI)** | **Matched Civilians**  **Median (95% CI)** | **Difference**  **Median (95% CI)** |
| --- | --- | --- | --- |
| **Initial, complex model (time and state fixed effects with 21 covariates)** | | | |
| High - Low HFR | | | |
| for All Suicide | 5.15 (1.69, 9.21) | 4.02 (0.94, 7.50) | 1.15 (-0.24, 2.67) |
| for Firearm Suicide | 6.43 (3.82, 9.51) | 6.26 (3.95, 9.00) | 0.17 (-1.10, 1.55) |
| for Nonfirearm Suicide | -1.28 (-2.24, -0.20) | -2.27 (-3.11, -1.37) | 0.98 (0.46, 1.50) |
| Restrictive - Permissive Law | | | |
| for All Suicide | -1.16 (-4.63, 2.13) | -1.99 (-5.15, 0.96) | 0.81 (-0.59, 2.20) |
| for Firearm Suicide | -1.51 (-3.93, 0.76) | -1.94 (-3.95, -0.03) | 0.41 (-0.82, 1.64) |
| for Nonfirearm Suicide | 0.37 (-0.86, 1.48) | -0.03 (-1.33, 1.13) | 0.42 (-0.32, 1.13) |
| **Final model (time and state random effects with 2 covariates)** | | | |
| High - Low HFR | | | |
| for All Suicide | 4.35 (1.90, 7.14) | 3.31 (1.11, 5.77) | 1.03 (-0.23, 2.43) |
| for Firearm Suicide | 5.85 (3.92, 8.01) | 5.77 (4.06, 7.66) | 0.08 (-1.16, 1.37) |
| for Nonfirearm Suicide | -1.49 (-2.18, -0.73) | -2.46 (-3.05, -1.78) | 0.96 (0.47, 1.43) |
| Restrictive - Permissive Law | | | |
| for All Suicide | -2.49 (-4.64, -0.21) | -3.19 (-5.22, -1.16) | 0.70 (-0.67, 2.03) |
| for Firearm Suicide | -2.44 (-4.00, -0.78) | -2.68 (-4.02, -1.36) | 0.26 (-0.93, 1.40) |
| for Nonfirearm Suicide | -0.06 (-0.87, 0.77) | -0.50 (-1.35, 0.35) | 0.45 (-0.26, 1.13) |

Note: Effects are the posterior median marginal effects expressed as suicide rates per 100,000, along with 95% credible intervals. *High-low HFR* is the difference in expected suicide rates associated between high and low state HFR defined by the 75^th^ and 25^th^ percentiles of HFR in 2017-2019. *Restrictive-permissive law* is the difference in expected suicide rates between restrictive and permissive state firearm laws, defined by the 75^th^ and 25^th^ percentiles of state law restrictiveness score in 2017-2019. Difference column reflects the difference in marginal effects between veteran and matched nonveteran populations.

**Table S3: Quantiles of the posterior distribution of final model parameters.**

| **Model parameter** | **2.5%** | **10%** | **Median** | **90%** | **97.5%** |
| --- | --- | --- | --- | --- | --- |
| Intercept | 2.21 | 2.24 | 2.28 | 2.32 | 2.35 |
| Firearm Method | 0.61 | 0.62 | 0.64 | 0.66 | 0.66 |
| Veteran | -0.17 | -0.15 | -0.12 | -0.09 | -0.08 |
| Veteran x Firearm | 0.03 | 0.05 | 0.09 | 0.12 | 0.14 |
| Veteran x Linear time | 0.01 | 0.02 | 0.03 | 0.03 | 0.04 |
| Veteran x Linear time x Firearm | -0.01 | 0.00 | 0.01 | 0.02 | 0.02 |
| HFR | -0.27 | -0.25 | -0.21 | -0.17 | -0.15 |
| HFR* x Firearm | 0.40 | 0.41 | 0.44 | 0.46 | 0.48 |
| HFR* x Veteran | 0.05 | 0.06 | 0.09 | 0.12 | 0.13 |
| HFR* x Veteran x Firearm | -0.17 | -0.16 | -0.12 | -0.08 | -0.06 |
| Law index* | -0.08 | -0.06 | -0.03 | 0.00 | 0.02 |
| Law index* x Firearm | -0.10 | -0.09 | -0.07 | -0.04 | -0.03 |
| Law index* x Veteran | -0.02 | 0.00 | 0.03 | 0.05 | 0.07 |
| Law index* x Veteran x Firearm | -0.06 | -0.04 | 0.00 | 0.04 | 0.05 |
| Percent black* | -0.09 | -0.08 | -0.05 | -0.03 | -0.01 |
| Population density* | -0.16 | -0.14 | -0.10 | -0.06 | -0.04 |
| Alabama | -1.12 | -0.84 | -0.36 | 0.10 | 0.37 |
| Alaska | -1.87 | -1.61 | -1.10 | -0.61 | -0.34 |
| Arizona | 0.35 | 0.55 | 0.95 | 1.36 | 1.58 |
| Arkansas | -0.57 | -0.35 | 0.04 | 0.42 | 0.62 |
| California | 0.06 | 0.31 | 0.76 | 1.23 | 1.50 |
| Colorado | 0.67 | 0.85 | 1.24 | 1.66 | 1.90 |
| Connecticut | -1.07 | -0.79 | -0.26 | 0.28 | 0.56 |
| Delaware | -0.44 | -0.19 | 0.33 | 0.82 | 1.09 |
| Florida | 1.46 | 1.70 | 2.13 | 2.58 | 2.82 |
| Georgia | -1.00 | -0.70 | -0.20 | 0.31 | 0.59 |
| Hawaii | -1.94 | -1.60 | -1.00 | -0.38 | -0.04 |
| Idaho | -0.24 | -0.03 | 0.40 | 0.84 | 1.06 |
| Illinois | -0.96 | -0.73 | -0.31 | 0.11 | 0.33 |
| Indiana | -0.71 | -0.49 | -0.13 | 0.26 | 0.45 |
| Iowa | -1.18 | -0.87 | -0.31 | 0.27 | 0.55 |
| Kansas | -1.16 | -0.94 | -0.53 | -0.15 | 0.07 |
| Kentucky | -0.67 | -0.44 | -0.06 | 0.33 | 0.56 |
| Louisiana | -1.54 | -1.22 | -0.65 | -0.10 | 0.19 |
| Maine | -1.03 | -0.81 | -0.38 | 0.04 | 0.25 |
| Maryland | 0.08 | 0.34 | 0.89 | 1.47 | 1.76 |
| Massachusetts | -2.04 | -1.73 | -1.11 | -0.50 | -0.19 |
| Michigan | -0.47 | -0.29 | 0.04 | 0.37 | 0.57 |
| Minnesota | -1.93 | -1.70 | -1.24 | -0.83 | -0.60 |
| Mississippi | -1.40 | -1.07 | -0.41 | 0.22 | 0.57 |
| Missouri | -0.39 | -0.20 | 0.15 | 0.51 | 0.70 |
| Montana | -0.14 | 0.14 | 0.67 | 1.21 | 1.50 |
| Nebraska | -2.35 | -2.04 | -1.47 | -0.93 | -0.64 |
| Nevada | 1.94 | 2.20 | 2.73 | 3.30 | 3.62 |
| New Hampshire | -0.61 | -0.38 | 0.05 | 0.51 | 0.73 |
| New Jersey | -0.91 | -0.59 | 0.04 | 0.70 | 1.04 |
| New Mexico | 0.46 | 0.69 | 1.15 | 1.63 | 1.91 |
| New York | -2.31 | -2.04 | -1.51 | -1.03 | -0.78 |
| North Carolina | -0.01 | 0.24 | 0.75 | 1.30 | 1.59 |
| North Dakota | -2.98 | -2.67 | -2.10 | -1.57 | -1.30 |
| Ohio | -0.64 | -0.42 | -0.04 | 0.36 | 0.57 |
| Oklahoma | -0.56 | -0.34 | 0.03 | 0.40 | 0.61 |
| Oregon | 0.44 | 0.67 | 1.05 | 1.47 | 1.70 |
| Pennsylvania | -0.22 | -0.01 | 0.37 | 0.75 | 0.96 |
| Rhode Island | -0.70 | -0.37 | 0.29 | 0.98 | 1.30 |
| South Carolina | -0.77 | -0.51 | -0.03 | 0.44 | 0.70 |
| South Dakota | -2.49 | -2.19 | -1.66 | -1.15 | -0.93 |
| Tennessee | -0.10 | 0.08 | 0.46 | 0.84 | 1.04 |
| Texas | -1.46 | -1.25 | -0.89 | -0.53 | -0.33 |
| Utah | 0.28 | 0.50 | 0.95 | 1.40 | 1.66 |
| Vermont | -0.66 | -0.40 | 0.10 | 0.60 | 0.86 |
| Virginia | -0.95 | -0.75 | -0.37 | 0.00 | 0.18 |
| Washington | -0.13 | 0.05 | 0.40 | 0.76 | 0.96 |
| West Virginia | -1.23 | -0.95 | -0.47 | 0.02 | 0.26 |
| Wisconsin | -0.99 | -0.78 | -0.40 | -0.03 | 0.19 |
| Wyoming | 0.08 | 0.33 | 0.86 | 1.40 | 1.72 |
| 2002-2004 | -1.54 | -1.18 | -0.51 | 0.09 | 0.39 |
| 2005-2007 | -2.27 | -1.86 | -1.05 | -0.38 | -0.06 |
| 2008-2010 | -1.31 | -0.98 | -0.35 | 0.24 | 0.57 |
| 2011-2013 | -0.80 | -0.47 | 0.11 | 0.70 | 1.02 |
| 2014-2016 | -0.21 | 0.12 | 0.77 | 1.49 | 1.95 |
| 2017-2019 | -0.01 | 0.35 | 1.05 | 1.83 | 2.28 |
| Inverse of dispersion parameter | 0.02 | 0.02 | 0.02 | 0.02 | 0.02 |
| Std. Dev. of state random effects | 0.11 | 0.12 | 0.13 | 0.16 | 0.17 |
| Std. Dev. of time random effects | 0.03 | 0.04 | 0.06 | 0.10 | 0.14 |

Note: * indicates a continuous state characteristics where the coefficients represent the effect associated with one SD change in the predictor, all other predictors are dichotomous 0,1. All coefficients are on a log Incident Risk Ratio scale.

**Table S4: Comparison of marginal effects for state law restrictiveness when pre-existing differences in state law restrictiveness are vs are not used to estimate these associations, United States, 2017-2019.**

| **Contrast**  **(greater vs less law restrictiveness)** | **With pre-existing state differences in law restrictiveness** | **Without pre-existing state differences in law restrictiveness** |
| --- | --- | --- |
| Total veteran suicides | -2.49 (-4.64, -0.21) | -2.68 (-8.12, 2.60) |
| Total nonveteran suicides | -3.19 (-5.22, -1.16) | 1.60 (-3.35, 6.38) |
| Veteran firearm suicides | -2.44 ( -4.00, -0.78) | -3.26 (-7.53, 1.33) |
| Nonveteran firearm suicides | -2.68 ( -4.02, -1.36) | -0.52 (-4.48, 3.32) |

Note: Effects are the posterior median marginal effects expressed as suicide rates per 100,000, along with 95% credible intervals. *With pre-existing state law differences* estimates are from our primary model which identifies effects with both across-time and across-state differences in laws. *Without pre-existing state law differences* estimates are from a model where effects are indicated only by changes in state laws within the study period.

**Table S5: Comparison of marginal effects for HFR when pre-existing differences in state law restrictiveness are vs are not used to estimate these associations, United States, 2017-2019.**

| **Contrast**  **(Higher vs lower HFR)** | **With pre-existing state differences in law restrictiveness** | **Without pre-existing state differences in law restrictiveness** |
| --- | --- | --- |
| Total veteran suicides | 5.15 (1.69, 9.21) | 1.54 (-4.30, 8.02) |
| Total nonveteran suicides | 4.02 (0.94, 7.50) | 4.96 (-0.57, 10.70) |
| Veteran firearm suicides | 6.43 (3.82, 9.51) | 2.78 (-2.07, 8.46) |
| Nonveteran firearm suicides | 6.26 (3.95, 9.00) | 4.86 (0.48, 9.81) |

Note: Effects are the posterior median marginal effects expressed as suicide rates per 100,000, along with 95% credible intervals. *With pre-existing state law differences* estimates are from our primary model which identifies effects with both across-time and across-state differences in laws. *Without pre-existing state law differences* estimates are from a model where effects are indicated only by changes in state laws within the study period.
